# A new quantum-inspired pattern based on Goldner-Harary graph for automated alzheimer’s disease detection

**DOI:** 10.1007/s11571-025-10249-7

**Published:** 2025-05-10

**Authors:** Ilknur Sercek, Niranjana Sampathila, Irem Tasci, Tuba Ekmekyapar, Burak Tasci, Prabal Datta Barua, Mehmet Baygin, Sengul Dogan, Turker Tuncer, Ru-San Tan, U. R. Acharya

**Affiliations:** 1 Department of Digital Forensics Engineering, College of Technology, Firat University, Elazig, Turkey; 2 Department of Biomedical Engineering, Manipal Academy of Higher Education, Manipal, India; 3 Department of Neurology, School of Medicine, Firat University, Elazig, 23119 Turkey; 4 Department of Neurology, Malatya Training and Research Hospital, 44000 Malatya, Turkey; 5 Vocational School of Technical Sciences, Firat University, 23119 Elazig, Turkey; 6 School of Business (Information System), University of Southern Queensland, Toowoomba, Australia; 7 Department of Computer Engineering, College of Engineering, Erzurum Technical University, Erzurum, Turkey; 8 Department of Cardiology, National Heart Centre Singapore, Singapore, Singapore; 9 Duke-NUS Medical School, Singapore, Singapore; 10 School of Mathematics, Physics and Computing, University of Southern Queensland, Springfield, Australia

**Keywords:** Goldner-Harary graph, GHPat, Alzheimer’s disease, EEG, Signal classification, Brain-computer interface

## Abstract

Alzheimer's disease (AD) is a common cause of dementia. We aimed to develop a computationally efficient yet accurate feature engineering model for AD detection based on electroencephalography (EEG) signal inputs. New method: We retrospectively analyzed the EEG records of 134 AD and 113 non-AD patients. To generate multilevel features, a multilevel discrete wavelet transform was used to decompose the input EEG-signals. We devised a novel quantum-inspired EEG-signal feature extraction function based on 7-distinct different subgraphs of the Goldner-Harary pattern (GHPat), and selectively assigned a specific subgraph, using a forward-forward distance-based fitness function, to each input EEG signal block for textural feature extraction. We extracted statistical features using standard statistical moments, which we then merged with the extracted textural features. Other model components were iterative neighborhood component analysis feature selection, standard shallow k-nearest neighbors, as well as iterative majority voting and greedy algorithm to generate additional voted prediction vectors and select the best overall model results. With leave-one-subject-out cross-validation (LOSO CV), our model attained 88.17% accuracy. Accuracy results stratified by channel lead placement and brain regions suggested P4 and the parietal region to be the most impactful. Comparison with existing methods: The proposed model outperforms existing methods by achieving higher accuracy with a computationally efficient quantum-inspired approach, ensuring robustness and generalizability. Cortex maps were generated that allowed visual correlation of channel-wise results with various brain regions, enhancing model explainability.

## Introduction

Alzheimer's disease (AD) is a common neurodegenerative cause of dementia; the prevalence is projected to triple in the next five decades (Rosende-Roca et al. 2025; Westphal Filho et al. 2025). The disease typically presents initially with mild cognitive impairment (MCI), which encompasses mild symptoms like memory loss but generally does not significantly impede daily functioning. Approximately 6–25% of individuals with MCI progress to AD annually (Barthélemy et al. 2024). They manifest symptoms of mild and moderate AD, with escalating cognitive deficits and decreased independence; and ultimately severe AD, with complete dependence on caregivers (Kwon et al. 2024). Even as definitive treatment for AD remains to be discovered, certain medications can delay symptom onset, underscoring the need for early diagnosis. The latter presents challenges as early symptoms are often mistakenly attributed to normal aging. A high index of suspicion is obligatory: suspect AD cases should undergo neuroimaging and laboratory examinations to exclude alternative neurological and systemic conditions, as well as neuropsychological assessment to confirm the diagnosis (Dauwels et al. 2010).

Electroencephalography (EEG), which records surface electrical signals emanating from brain activity, is altered in MCI and AD (Sasidharan et al. 2025). In AD, EEG brain signals or waves may demonstrate slowdown, reduced complexity, and disrupted synchronization (Arjmandi-Rad et al. 2024). Moreover, there is a strong correlation between visually-assessed EEG scores and the severity of dementia as assessed by the Mini-Mental State Examination (Keresztes et al. 2024; Fan et al. 2024). Loss of posterior dominant alpha rhythm and diffuse EEG slowing have also been reported in patients with confirmed AD (Chu 2024). Compared with age-matched healthy controls, both MCI and AD are associated with EEG signal slowing (Meghdadi et al. 2024), decreased power in the alpha and beta frequency bands (8–30 Hz), and increased power in the delta and theta (0.5–8 Hz) and gamma (30–100 Hz) frequency bands (Ettenberger et al. 2024).

Expert interpretation of the EEG is the standard of care but is time-intensive and subject to human biases (Dutta et al. 2024). In addition, reproducible detection of AD-induced EEC signal perturbations is confounded by substantial variability in EEG findings among AD patients. Consequently, none of the above-mentioned AD-associated EEG phenomena provides a reliable clinical diagnosis of early-stage AD. Advancements in signal processing and machine learning have spurred the development of automated EEG-based models for AD detection that may surmount the limitations of manual EEG interpretation. In parallel, EEG recording systems, including portable wearable ones (Rehman et al. 2024), have become available and are relatively affordable. These trends make EEG an accessible and promising tool for MCI/AD screening and early detection among high-risk populations (Dauwels et al. 2010). In the current study, we proposed an automated model for AD detection based on a novel quantum-inspired EEG signal feature extraction function.

### Literature review

There are various studies in the literature for different disciplines (Abedinzadeh Torghabeh et al. 2023; Hakemi et al. 2024). In recent years, several machine learning classification models for EEG-based AD diagnosis have been published (Table 1). Some studies focused on deep learning techniques (Bi and Wang 2019; Fouladi et al. 2022; Lopes and Cassani 2023; Xia et al. 2023), in which high classification performance came at a cost of high computational complexity (Lopes and Cassani 2023). Moreover, data augmentation was used in Xia et al. (2023). Data augmentation can lead to unreliable results and overfitting. Lastly, in some models, the segmentation times were very short (Bi and Wang 2019; Rodrigues et al. 2021; Chedid et al. 2022), which could potentially result in overfitting.

**Table 1:** Summary of selected machine learning-based studies developed for automated detection of AD using EEG signals

Author(s)	Dataset Features	Methods	Acc (%)	Limitations
Bi and Wang (2019)	Collected dataset, 3 classes (4 HC, 4 MCI, 4 AD), 64 channels, 0.5-s segments	EEG image conversion with spectral topography maps, contractive slab, and spike convolutional deep Boltzmann machine	95.04	High computational complexity, and low segmentation rate
Rodrigues et al. (2021)	Collected dataset, 4 classes (11 HC, 8 MCI, 11 MAD and 8 AD), 19 channels, 5-s segments	Discrete wavelet transform, cepstral and lacstral analyses, feature normalization, and artificial neural network	95.55	Fewer subjects, small sample size, and low segmentation rate
Safi and Safi (2021)	Public dataset, 3 classes (35 HC, 31 MAD, 20 AD), 20 channels, 8-s segments	Power spectral density, empirical mode decomposition, discrete wavelet transform, Hjorth parameters, and kNN	97.64	Fewer subjects, small sample size, and low segmentation rate
Dogan et al. (2022)	Public dataset, 2 classes (11 HC, 12 AD), 16 channels, 663 EEG segments	Primate brain pattern, iterative neighborhood component analysis, kNN, and iterative majority voting	92.01	Fewer subjects, and a small number of EEG segments
Pirrone et al. (2022)	Public dataset, 3 classes (23 HC, 37 MCI, 49 AD), 19 channels, 105 EEG records	Power spectral density, double digital filter, and kNN	86.0	Fewer EEG records, and relatively low accuracy
Fouladi et al. (2022)	Public dataset, 3 classes (61 HC, 56 MCI, 63 AD), 19 channels, 2-s segments	Continuous wavelet transform, and CNN	92.70	High computational complexity, and low segmentation rate
Chedid et al. (2022)	Collected dataset, 2 classes (23 HC, 20 AD), 32 channels, 1-s segments	Band-wise power spectral density, statistical analysis, and logistic regression	81.11	Small number of subjects, and low segmentation rate
Xia et al. (2023)	Public dataset, 3 classes (14 HC, 37 MCI, 49 AD), 19 channels	Fast Fourier transform, data augmentation, and deep pyramid CNN	97.10	Overlapping segmentation, data augmentation, and high computational complexity
Lopes and Cassani (2023)	Collected dataset, 3 classes (20 HC, 19 MAD, 15 AD), 20 channels, 8-s segments	CNN, saliency map extraction, ANOVA-F value feature selection, and support vector machine	90.50	Overlapping segmentation with 1-s, relatively low accuracy, and high computational complexity
El-Assy et al. (2024)	Public dataset, 5 classes (171 AD, 72 LMCI, 233 MCI, 240 EMCI, 580 CN)	CNN	99.57	High computational complexity
Abuhantash et al. (2024)	Public dataset (ADNI 1331 participants and AIBL 858 participants)	GNN	99.00	High computational complexity
Zarei et al. (2024)	Public dataset, 3 classes (199 AD, 200 MCI, 200 CN)	CNN	84.4	High computational complexity
Kim et al. (2024)	Collected dataset (Resting State EEG, Memory Encoding EEG)	cKNN	93.10	Relatively small number of subjects
Dogan et al. (2024)	Public dataset, 2 classes, (12 AD, 11 HC), 59 channels, 15-s segments	Lattice123 pattern, Multilevel discrete wavelet transform	99.62	Small number of subjects
Ohal and Mantri (2024)	Public dataset, 2 classes, (11 MCI, 16 HC)	Statistical analysis	92	Relatively small number of subjects
Siuly et al. (2024)	Public dataset, 3 classes, (31 mild AD, 20 moderate AD, 35 HC), 16-s non-overlapping segments	Long short-term memory	99.00	High computational complexity
Rezaee and Zhu (2025)	Public dataset, 2 classes, (59 AD, 56 HC), 21 channels	Discrete wavelet transform, improved CascadeNet model	98.84	High computational complexity
Nour et al. (2024)	Public dataset, 2 classes, (24 AD, 24 HC), 19 channels	2-dimensional CNN	97.90	High computational complexity
Le and Nguyen (2024)	Public dataset, 3 classes, (36 AD, 23 FTD, 29 HC)	CNN	87.30 for FD vs. HC	High computational complexity
Sen et al. (2024)	Public dataset, 2 classes, (15 AD, 11 HC), 19 channels, 5-s segments	1-dimensional CNN	94.00	High computational complexity, and low segmentation rate
Sharma and Meena (2024)	Public dataset, 2 classes, (24 AD, 24 HC)	Graph Fourier transform, discrete wavelet transform	98.9	Relatively small number of subjects

^**^Acc: accuracy; AD: Alzheimer’s disease; CNN: convolutional neural network; HC: healthy control; kNN: k-nearest neighbors; MAD: mild Alzheimer’s disease; MCI: mild cognitive impairment; EMCI: early mild cognitive impairment; LMCI: late mild cognitive impairment: GNN, graph neural network; cKNN, correlation-based k-nearest neighbors; FD, frontotemporal dementia

### Literature gap

From the studies in Table 1, we observed the following:

1. Fewer number of participants or EEG samples. Existing AD-related EEG signal datasets contain few participants, which reduces the generalizability of the study outcomes and results.
2. Suboptimal validation methodology. Machine learning models commonly rely on random separation-based validation approaches, which may lead to unreliable classification results.
3. Superior performance of deep learning models. The use of backpropagation and forward algorithms enables deep models to select the most suitable weights for the networks. In contrast, feature engineering models rely on fixed feature selectors, which may not identify the most appropriate features consistently.
4. Quantum mechanism is a very popular research area since quantum models provide many advantages and these advantages have been utilized in feature engineering (Li et al. 2020; Safriandono et al. 2024). For instance, lattice-based feature extractors are quantum-inspired feature extraction functions. However, there are limited quantum-based feature engineering models.

### Motivation and our method

We were motivated to develop a computationally efficient yet accurate feature engineering model for AD detection. To generate multilevel features akin to deep learning models, the multilevel discrete wavelet transform (MDWT) (Desai and Sankhe 2012) was used to decompose the input EEG signals. Based on the concept of superposition in quantum physics, in which a quantum system is able to exist in multiple states at the same time until it is measured, we devised a novel EEG signal feature extraction function based on different subgraphs derivable from the Goldner-Harary (GH) graph (Swain 2019; Swain et al. 2020). A straightforward forward-forward (FF) distance-based fitness function inspired by Hinton's FF algorithm (Hinton 2022) was employed to assign a distinct subgraph (analogous to “quantum state”) to each overlapping block of EEG signal input for the local binary pattern (LBP)-like (Ojala et al. 2002) textural feature extraction. This FF strategy mimicked human neural systems more realistically, and required shorter training times, compared with the backpropagation (which is unlikely to transpire in nature (Hinton 2022)) commonly used in deep models. In parallel, we extracted statistical features using standard statistical moments, which we then merged with the extracted textural features. Other model components were iterative neighborhood component analysis (INCA) feature selection (Tuncer et al. 2020), standard shallow k-nearest neighbors (kNN) classification (Peterson 2009), as well as iterative majority voting (IMV) (Dogan et al. 2021) and greedy algorithm to generate additional voted prediction vectors and select the best overall model results, respectively. Adopting a leave-one-subject-out cross-validation (LOSO CV) strategy, our GH graph pattern-based model, GHPat, was trained and validated on a large 20-channel AD EEG signal dataset. Our proposed GHPat is a handcrafted feature extraction model by inspiring a quantum mechanism since there are seven feature extraction functions in this method and by selecting the best feature vector using a Hinton’s FF (Hinton 2022) method, like a feature vector selection method.

### Innovations and contributions

Novelties and contributions of our research include:

Novelties:

1. A novel quantum-inspired graph-based feature extraction function was incorporated into a computationally efficient feature engineering model for AD detection. In this regard, this work presents an innovative feature extractor that is quantum-inspired and by using this quantum-inspired feature extractor, a quantum feature engineering model has been presented.
2. A large training EEG signal dataset was collected from AD patients and non-AD controls.

Contributions

1. Many existing machine learning EEG-based models for AD detection are limited by relatively small datasets. The results of the GHPat model trained on our large EEG dataset would be more generalizable in comparison.
2. Compared with standard validation techniques based on random separation, the LOSO CV strategy used in our study accounted for inter-subject differences, which better simulate real-life diagnostic applications in individual patients. Hence, the results of our model were arguably more reliable.
3. The model incorporated IMV and greedy algorithm for automatically selecting the most accurate model results, which rendered the GHPat feature engineering model fully self-organized.
4. The GHPat model attained a good 88.17% classification accuracy. Further, we could correlate channel-wise results with the underlying spatial brain cortical maps to provide explainable results, which enhanced the interpretability of our model.
5. To the best of my knowledge, we are the first group to use such a huge database and propose a new feature engineering model and report high classification accuracy of 88.17% with a leave- one- subject- out (LOSO) cross-validation strategy.

## Dataset

We retrospectively analyzed the EEG records of 134 AD (mean age 78.51 ± 7.53 years; 76 male, 58 female) and 113 non-AD (mean age 74.44 ± 8.50 years; 59 male, 54 female) patients (total number 247; age range 50 to 99 years) and had their diagnoses confirmed by a neurologist. The retrospective collection and analysis of the EEG records had been approved by the institutional review board. Each EEG record comprised 20 channels: Channels 1 to 20 were (in order) Fp1; Fp2; F3; F4; C3; C4; P3; P4; O1; O2; F7; F8; T3; T4; T5; T6; A1; A2; Fz; and Cz. The letters A, F, Fp, C, T, P, and O referred to the anterior, frontal, prefrontal, central, temporal, parietal, and occipital positions of the scalp electrodes that overlie the corresponding brain regions; and z referred to mid-sagittal electrode placement. The numbers increased with distance from the midline; even and odd numbers represented left- and right-brain channels, respectively. The EEG signals were sampled at 500 Hz and were divided into non-overlapping 15-s segments for analysis, i.e., segment data length of 7500. The dataset comprised 6857 EEG segments, with 2948 and 3909 segments in the AD and control classes, respectively.

## The presented quantum-inspired graph pattern

**Fig. 1:**
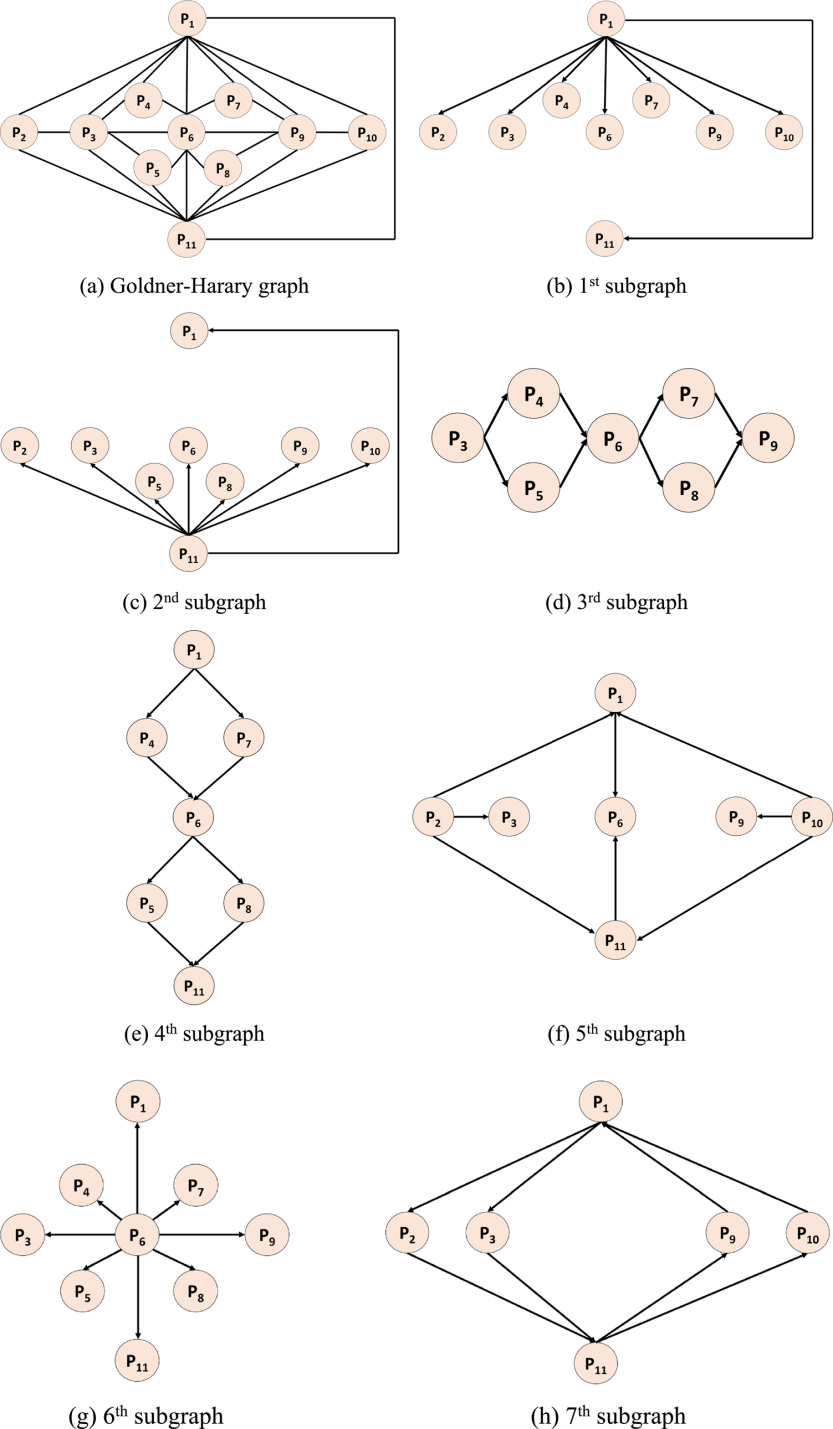
Non-directed Goldner-Harary graphs: **a** contains eleven numbered nodes (P) and 27 non-directed edges. The seven derived subgraphs (b to h) each contain a variable number (six, seven, or nine) of nodes and eight directed edges (arrows). The directions (given by pairs of P numbers within the initial and terminal nodes) and order of the directed edges determine the matrix used for extracting textural feature bits from each fixed-length overlapping signal block, which in turn had been partitioned from the input EEG segment

The GH graph, the smallest non-Hamiltonian maximal planar graph, comprises 11 nodes and 27 non-directed edges (Fig. 1a) (Swain 2019; Swain et al. 2020). Analogous to the concept of superposition in quantum mechanics, we defined seven distinct directed subgraphs (Fig. 1b–h) for graph pattern-based LBP-like textural feature extraction. Each input EEG signal segment was first partitioned into overlapping fixed-length signal blocks; the most optimal subgraph pattern for each block was chosen using a selection function inspired by Hinton’s FF algorithm. Knowledge of the specific dataset was imperative for making an informed subgraph selection: this mirrors the principle of quantum uncertainty, where the quantum state has multiple possibilities that only materialize as one at the time of measurement.

The generation of seven-bit feature vectors was accomplished using the matrix determined by the directed edges within each of the seven subgraphs in conjunction with the signum function, $sgn(.)$. Matrices corresponding to Subgraphs 1 to 7 are encompassed in order in Eqs. (1)-(7) below.

1 $$\left[\begin{array}{c}bi{t}_{1}^{1}\\ bi{t}_{2}^{1}\\ bi{t}_{3}^{1}\\ bi{t}_{4}^{1}\\ bi{t}_{5}^{1}\\ bi{t}_{6}^{1}\\ bi{t}_{7}^{1}\\ bi{t}_{8}^{1}\end{array}\right]=sgn\left(\left[\begin{array}{c} {P}_{1},{P}_{2}\\ {P}_{1},{P}_{3}\\ {P}_{1},{P}_{4}\\ {P}_{1},{P}_{6}\\ {P}_{1},{P}_{7}\\ {P}_{1},{P}_{9}\\ {P}_{1},{P}_{10}\\ {P}_{1},{P}_{11}\end{array}\right]\right)$$

2 $$\left[\begin{array}{c}bi{t}_{1}^{2}\\ bi{t}_{2}^{2}\\ bi{t}_{3}^{2}\\ bi{t}_{4}^{2}\\ bi{t}_{5}^{2}\\ bi{t}_{6}^{2}\\ bi{t}_{7}^{2}\\ bi{t}_{8}^{2}\end{array}\right]=sgn\left(\left[\begin{array}{c} {P}_{11},{P}_{2}\\ {P}_{11},{P}_{3}\\ {P}_{11},{P}_{5}\\ {P}_{11},{P}_{6}\\ {P}_{11},{P}_{8}\\ {P}_{11},{P}_{9}\\ {P}_{11},{P}_{10}\\ {P}_{11},{P}_{1}\end{array}\right]\right)$$

3 $$\left[\begin{array}{c}bi{t}_{1}^{3}\\ bi{t}_{2}^{3}\\ bi{t}_{3}^{3}\\ bi{t}_{4}^{3}\\ bi{t}_{5}^{3}\\ bi{t}_{6}^{3}\\ bi{t}_{7}^{3}\\ bi{t}_{8}^{3}\end{array}\right]=sgn\left(\left[\begin{array}{c} {P}_{3},{P}_{4}\\ {P}_{3},{P}_{5}\\ {P}_{4},{P}_{6}\\ {P}_{5},{P}_{6}\\ {P}_{6},{P}_{7}\\ {P}_{6},{P}_{8}\\ {P}_{7},{P}_{9}\\ {P}_{8},{P}_{9}\end{array}\right]\right)$$

4 $$\left[\begin{array}{c}bi{t}_{1}^{4}\\ bi{t}_{2}^{4}\\ bi{t}_{3}^{4}\\ bi{t}_{4}^{4}\\ bi{t}_{5}^{4}\\ bi{t}_{6}^{4}\\ bi{t}_{7}^{4}\\ bi{t}_{8}^{4}\end{array}\right]=sgn\left(\left[\begin{array}{c} {P}_{1},{P}_{4}\\ {P}_{1},{P}_{7}\\ {P}_{4},{P}_{6}\\ {P}_{7},{P}_{6}\\ {P}_{6},{P}_{5}\\ {P}_{6},{P}_{8}\\ {P}_{5},{P}_{11}\\ {P}_{8},{P}_{11}\end{array}\right]\right)$$

5 $$\left[\begin{array}{c}bi{t}_{1}^{5}\\ bi{t}_{2}^{5}\\ bi{t}_{3}^{5}\\ bi{t}_{4}^{5}\\ bi{t}_{5}^{5}\\ bi{t}_{6}^{5}\\ bi{t}_{7}^{5}\\ bi{t}_{8}^{5}\end{array}\right]=sgn\left(\left[\begin{array}{c} {P}_{2},{P}_{1}\\ {P}_{2},{P}_{3}\\ {P}_{2},{P}_{11}\\ {P}_{1},{P}_{6}\\ {P}_{11},{P}_{6}\\ {P}_{10},{P}_{9}\\ {P}_{10},{P}_{1}\\ {P}_{10},{P}_{11}\end{array}\right]\right)$$

6 $$\left[\begin{array}{c}bi{t}_{1}^{6}\\ bi{t}_{2}^{6}\\ bi{t}_{3}^{6}\\ bi{t}_{4}^{6}\\ bi{t}_{5}^{6}\\ bi{t}_{6}^{6}\\ bi{t}_{7}^{6}\\ bi{t}_{8}^{6}\end{array}\right]=sgn\left(\left[\begin{array}{c} {P}_{6},{P}_{1}\\ {P}_{6},{P}_{3}\\ {P}_{6},{P}_{11}\\ {P}_{6},{P}_{9}\\ {P}_{6},{P}_{4}\\ {P}_{6},{P}_{7}\\ {P}_{6},{P}_{8}\\ {P}_{6},{P}_{5}\end{array}\right]\right)$$

7 $$\left[\begin{array}{c}bi{t}_{1}^{7}\\ bi{t}_{2}^{7}\\ bi{t}_{3}^{7}\\ bi{t}_{4}^{7}\\ bi{t}_{5}^{7}\\ bi{t}_{6}^{7}\\ bi{t}_{7}^{7}\\ bi{t}_{8}^{7}\end{array}\right]=sgn\left(\left[\begin{array}{c} {P}_{1},{P}_{2}\\ {P}_{2},{P}_{11}\\ {P}_{11},{P}_{10}\\ {P}_{10},{P}_{1}\\ {P}_{1},{P}_{3}\\ {P}_{3},{P}_{11}\\ {P}_{11},{P}_{9}\\ {P}_{9},{P}_{1}\end{array}\right]\right)$$

To extract these bits, we have used an overlapping block with a length of 11 and the creation of this block is mathematically defined in Equation 8.

8 $${P}_{i}=signa{l}_{i+j-1}, i\in \left\{\text{1,2},\dots \xi -10\right\}, j\in \left\{\text{1,2},\dots ,11\right\}$$

By using the values of the overlapping block, Eqs. 1–7 and the signum function, binary features have been created and the mathematical illustration of the signum function is defined in Eq. 9.

9 $$sgn\left({P}_{1},{P}_{2}\right)=\left\{\begin{array}{c}0,{P}_{1}-{P}_{2}<0\\ 1,{P}_{1}-{P}_{2}\ge 0\end{array}\right.$$

**Fig. 2:**
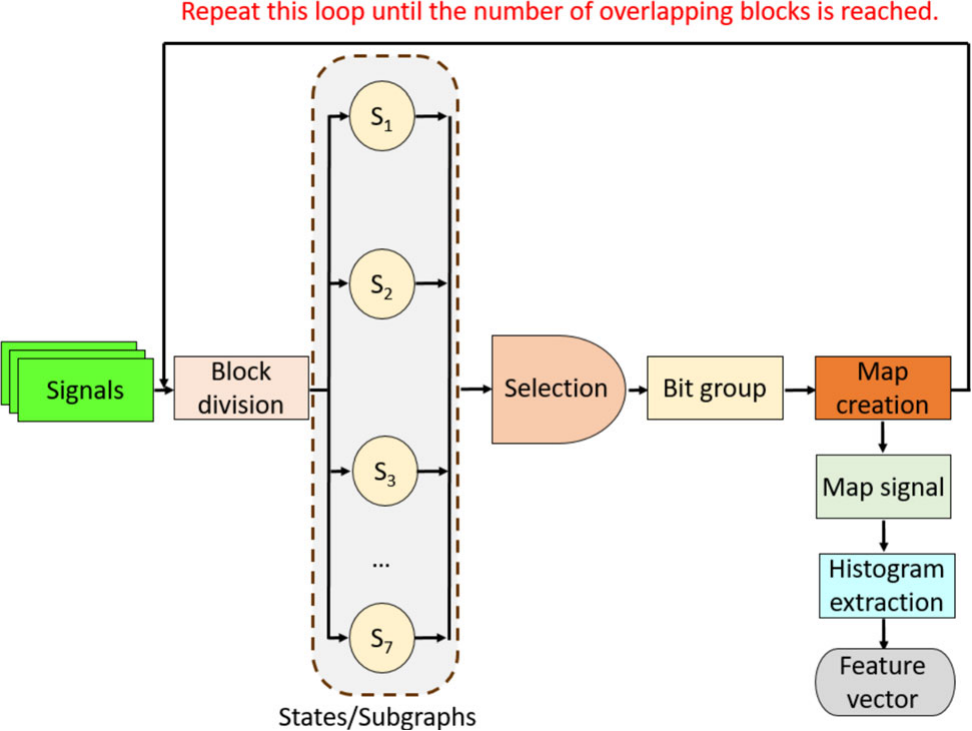
Illustration of textural feature extraction using the GHPat

In Eqs. (1)-(9), $bit$ represents binary features; $P$, the created overlapping block of length 11 (as the GH graph has 11 nodes); $signal$, the one-dimensional (1D) signal; and $\xi$, length of the signal. Using the generated bits, a feature map signal was constructed, from the histogram of which, the feature vector was derived (Fig. 2).

The steps of the GHPat-based feature extraction procedure are detailed below.

1: Generate overlapping blocks of length 11 from the input EEG signal segment.

2: Calculate the average value of the signal.

10 $$\mu =\frac{1}{\xi }\sum_{k}^{\xi }signa{l}_{k}$$

where $\mu$ represents the average value of the signal.

3: Compute the average value of used values in the subgraphs.

11 $$\begin{gathered} \mu_{1} = \frac{{P_{1} + P_{2} + P_{3} + P_{4} + P_{6} + P_{7} + P_{9} + P_{10} + P_{11} }}{9} \hfill \\ \mu_{2} = \frac{{P_{1} + P_{2} + P_{3} + P_{5} + P_{6} + P_{8} + P_{9} + P_{10} + P_{11} }}{9} \hfill \\ \mu_{3} = \frac{{P_{1} + P_{2} + P_{3} + P_{4} + P_{6} + P_{7} + P_{9} + P_{10} + P_{11} }}{9} \hfill \\ \mu_{4} = \frac{{P_{3} + P_{4} + P_{5} + P_{6} + P_{7} + P_{8} + P_{9} }}{7} \hfill \\ \mu_{5} = \frac{{P_{1} + P_{4} + P_{5} + P_{6} + P_{7} + P_{8} + P_{11} }}{7} \hfill \\ \mu_{6} = \frac{{P_{1} + P_{2} + P_{3} + P_{6} + P_{9} + P_{10} + P_{11} }}{7} \hfill \\ \mu_{7} = \frac{{P_{1} + P_{3} + P_{4} + P_{5} + P_{6} + P_{7} + P_{8} + P_{9} + P_{11} }}{9} \hfill \\ \end{gathered}$$

where ${\mu }_{1}, {\mu }_{2},\dots ,{\mu }_{7}$ represent the average values of the used subgraphs.

4: Calculate distances of the computed local average values to the average value of the signal (global average value). Here, we used the L1-norm distance metric to compute the distances of the average values.

12 $$dis{t}_{h}=\left|\mu -{\mu }_{h}\right|,h\in \left\{\text{1,2},\dots ,7\right\}$$

where $dist$ represents the distances of the average values.

5: Select a subgraph with the minimum distance to generate features.

13 $$ix=\text{min}(dist)$$

where $ix$ represents the index of the minimum distance.

6: Use the selected subgraph to generate bits. Steps 4 to 6 constitute the feedforward algorithm for selecting a specific subgraph for each input signal block.

7: Convert bit values to decimal values to generate feature map values.

14 $$f{m}_{i}=\sum_{q=1}^{8}bi{t}_{q}^{ix}\times {2}^{q-1}$$

where $bi{t}^{ix}$ represents binary features of the selected subgraph; and $fm$, feature map signal.

8: Repeat Steps 1–7 until the number of overlapping blocks is reached and generate the feature map signal.

9: Extract the histogram of the created feature map signal.

15 $$feature=\delta (fm)$$

where $feature$ represents feature vector; and $\delta (.)$, the histogram extraction function. In this step, we have extracted a feature vector with length 256 (= 2^8^), since each subgraph has eight edges. The above nine steps define our proposed GHPat-based feature extraction function.

## Proposed feature engineering model

**Fig. 3:**
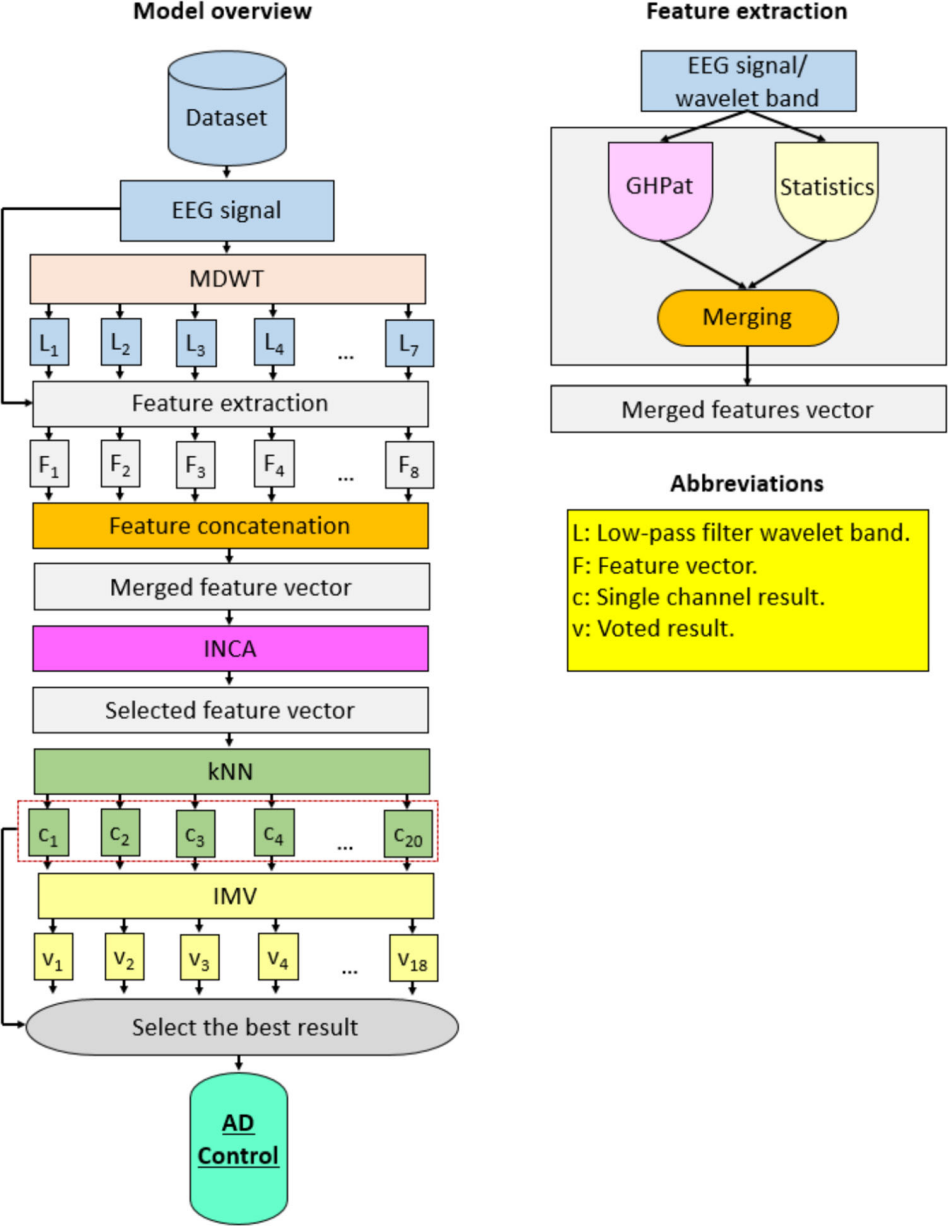
Proposed architecture of the GHPat-based Alzheimer’s disease classification model **AD, Alzheimer’s disease; c, channel-wise result; F, feature; IMV, iterative majority voting; INCA, iterative neighborhood component analysis; kNN, k-nearest neighbors; L, level; v, voted result.

Our model comprised four phases: multilevel GHPat and statistics-based feature extraction, feature selection, classification, and post-processing/information fusion (Fig. 3). To simulate multilevel/multilayered feature extraction in deep learning models, we used MDWT to decompose the EEG input signal to feed to downstream feature extractors, thereby enabling the generation of multilevel features. We extracted GHPat-based textural features and statistical features in parallel, which were then merged. In the feature selection phase, we used the INCA feature selector to find the best feature combination to feed to the established distance-based kNN classifier. Using LOSO CV, 20 channel-wise kNN-classified results were generated. In the post-processing phase, additional voted results were generated using IMV; and the most accurate result was selected using a greedy algorithm.

### Feature extraction

We applied seven-level MDWT (Desai and Sankhe 2012) to decompose the EEG signal using the symlet 4 mother wavelet function, and a low-pass filter, to extract seven low-pass wavelet bands. The wavelet bands generated and the raw signal have been utilized as input for the feature extraction functions and we have used two feature extractors. These are the proposed GHPat and the statistical feature extractor. In parallel to textural feature extraction (detailed in Sect. "The presented quantum-inspired graph pattern" above), we extracted statistical features using 14 standard statistical moments: average, maximum, minimum, median, standard deviation, kurtosis, skewness, range (maximum-minimum), root mean square, maximum absolute deviation, Shannon entropy, sure entropy, Tsallis entropy, and log entropy. From each raw EEG signal or wavelet band input, the 256 textural and 14 statistical features extracted by GHPat and statistical moments were merged to form a feature vector of 270 (= 256 + 14). Further, the eight generated feature vectors (1 from the raw EEG signal; 7 from the wavelet bands) were again merged to obtain a final feature vector of length 2160 (= 270 × 8). The steps are detailed below.

Step 1: Apply MDWT to the input raw EEG signal.

16 $$\left[lo{w}^{1}, hig{h}^{1}\right]=\psi (signal)$$

17 $$\left[lo{w}^{k+1}, hig{h}^{k+1}\right]=\psi \left(lo{w}^{k}\right), k\in \left\{\text{1,2},\dots ,6\right\}$$

where $low$ represents low pass filter coefficients; $high$, high pass filter coefficients; and $\psi (.)$, discrete wavelet transform function.

Step 2: Generate features from low pass filter bands and raw EEG signal.

18 $$fea{t}^{1}=\omega \left(\zeta \left(signal\right),\rho \left(signal\right)\right)$$

19 $$fea{t}^{h+1}=\omega \left(\zeta \left(lo{w}^{h}\right),\rho \left(lo{w}^{h}\right)\right), h\in \{\text{1,2},\dots ,7\}$$

where $feat$ represents feature vector of length 270 (= 256 + 14); $\zeta (.)$, GHPat feature extraction function; $\rho (.)$, defines statistical feature extraction function; and $\omega (.)$, concatenation function.

Step 3: Merge all eight generated feature vectors into one final feature vector.

20 $$final=\omega \left(fea{t}^{1},fea{t}^{2},\dots ,fea{t}^{8}\right)$$

where $final$ represents the final feature vector of length (= 270 × 8).

### Feature selection

To select the most discriminative features from 2160 generated in the feature extraction phase, we used INCA (Tuncer et al. 2020), a parametric feature selector with a range of iteration is 50 to 550, i.e. 501 (= 550–50 + 1) features in each selected feature vector; and loss value calculator, kNN classifier.

Step 4: Calculate sorted/qualified indexes of the final feature vector using neighborhood component analysis.

21 $$index=\pi \left(final,y\right)$$

where $index$ represents the sorted indexes; $\pi (.)$, the neighborhood component analysis feature selection function; and $y$, the actual output.

Step 5: Apply iterative feature vector selection.

22 $$\begin{gathered} sel^{h} \left( {d,g} \right) = final\left( {d,index\left( g \right)} \right),d \in \left\{ {1,2, \ldots ,nos} \right\}, \hfill \\ g \in \left\{ {50,51, \ldots ,550} \right\},h \in \left\{ {1,2, \ldots ,501} \right\} \hfill \\ \end{gathered}$$

where $sel$ represents the selected feature vector; and $nos$, the number of observations.

Step 6: Calculate the loss/misclassification rates of the selected feature vectors using the kNN classifier.

23 $$mcr\left(h\right)=knn\left(se{l}^{h}\right)$$

where $mcr$ represents the misclassification rate.

Step 7: Select the best-selected feature vector with the minimum misclassification/loss rate.

24 $$idx=\text{min}(loss)$$

25 $$select=se{l}^{idx}$$

where $idx$ represents the index of the minimum loss value; and $select$, the selected feature vector.

### Classification

We deployed a distance-based kNN classifier (Peterson 2009) with LOSO CV to generate channel-wise results. In contrast to random splitting of training/validation datasets, LOSO CV accounts for patient-based data segregation and arguably yields more reliable results. Here, we used weighted kNN with the following parameter settings: k, 10; distance, L1-norm (Manhattan distance); voting scheme, squared inverse.

Step 8: Generate the channel-wise results using the kNN classifier.

26 $$result=knn\left(select\right)$$

As each EEG record contained 20 channels, steps 1 to 8 were run 20 times to obtain 20 channel-wise results.

Step 9: Repeat steps 1–8 until a total number of channels is reached.

### Post-processing

We used IMV (Dogan et al. 2021), a parametric information fusion algorithm, to generate additional voted results. Within the IMV framework, we iterated from 3 to 20 to generate 18 (= 20–3 + 1) voted results using the mode function. Merging these with the 20 kNN-classified channel-wise results, we obtained a predicted label vector comprising 38 (= 20 + 18) elements. Finally, the greedy algorithm was used to identify the most accurate result. The post-processing (information fusion) steps are detailed below.

Step 10: Create voted vectors using the IMV algorithm.

27 $$vote=imv\left(result\right)$$

where $vote$ represents voted results; and ($imv$), IMV function.

Step 11: Calculate classification accuracies of the single channeled results and voted results.

28 $$ca{c}^{z}=\varphi \left({result}^{z},y\right), z\in \{\text{1,2},\dots ,20\}$$

29 $$ca{c}^{z+r}=\varphi \left({vote}^{r},y\right), r\in \{\text{1,2},\dots ,18\}$$

where $cac$ represents the calculated classification accuracies of the 20 kNN-classified and IMV-voted results.

Step 12: Choose the overall most accurate result for the model.

30 $$ind=\text{max}(acc)$$

31 $$ultimate=\left\{\begin{array}{c}resul{t}^{ind},ind\le 20\\ {vote}^{ind-20},ind>20\end{array}\right.$$

where $ind$ represents the index of maximum accuracy. The automatically run greedy algorithm helped transformed our model into a fully self-organized model.

## Experimental results

### Setup

The proposed architecture comprising the MDWT, GHPat, statistical feature extractor, INCA, kNN, IMV, and greedy algorithm (Table 2) is computationally lightweight, obviating the need for intensive hyperparameter tuning. We implemented the model on a CPU computer with a 3.6 GHz processor, 64 GB memory, and Windows 11 operating system, using MATLAB version-2023a programming environment and custom m files. The utilized functions were stored as m files to implement the recommended model. As the standard MATLAB classification learner toolbox did not possess LOSO CV functionality, we coded the kNN classifier ourselves.

**Table 2:** Details of GHPat model architecture

Method	Parameters	Input	Output
MDWT	Number of levels: 7; filter: symlet 4	1D signal	7 low pass filter bands
GHPat	Patterns: 7 subgraphs of the GH graph; kernel: signum; pattern selection: differences of means-based fitness function; length of the feature: 256	1D signal	256 features
Statistical feature extractor	14 statistical moments	1D signal	14 features
Multilevel feature extraction	MDWT + GHPat + statistical feature extractor	1D signal and 7 wavelet bands	8 feature vectors, each of length 270
Feature merging	Concatenation function	1D signal	Final feature vector of length 2160
INCA	Loop range: 50–550; loss function calculator: kNN; neighborhood component analysis: default setting, number of loops being half the number of observations	Feature vector	Most discriminative *n* features selected for each channel
kNN	k:10; distance: Manhattan; voting: squared inverse; validation: LOSO CV	Selected feature vectors	Channel-wise results (20 results)
IMV	Loop range: 3–20; sorting criteria: accuracy; majority function: mode	20 channel-wise results	18 voted results
Greedy algorithm	Select the most accurate result	20 channel-wise and 18 voted results	1 result with the maximum accuracy

### Results

We used two performance evaluation metrics, classification accuracy and geometric mean, which are calculated as shown below.

32 $$cac=\frac{TP+TN}{TP+TN+FN+FP}$$

33 $$gm=\sqrt{\frac{TP}{TP+FN}\times \frac{TN}{TN+FP}}$$

where $cac$ represents calculated accuracy; $gm$, geometric mean; and $TP$, $TN$, $FN$, and $FP$, the numbers of true positives, true negatives, false negatives, and false positives, respectively.

#### Channel-wise results

Using LOSO CV, the most and least accurate kNN-classified channel-wise results were observed in Channel 8 (P4) and Channel 6 (C4), respectively (Table 3).

**Table 3:** Channel-wise results obtained using the GHPat-based AD detection model

No	Name	Accuracy (%)	Geometric mean (%)	No	Name	Accuracy (%)	Geometric mean (%)
1	Fp1	72.09	71.25	11	F7	74.42	72.58
2	Fp2	69.65	69.19	12	F8	73.11	72.23
3	F3	74.73	73.24	13	T3	75.81	74.34
4	F4	74.10	72.96	14	T4	69.84	69
5	C3	78.10	76.44	15	T5	74.06	73.19
6	C4	64.65	63.29	16	T6	71.26	70.82
7	P3	77.50	76.36	17	A1	74.86	73.93
8	P4	**78.15**	**77.46**	18	A2	69.96	69.70
9	O1	76.65	75.36	19	Fz	71.24	70.71
10	O2	74.86	74.06	20	Cz	67.33	66.43

#### Information fusion results

**Fig. 4:**
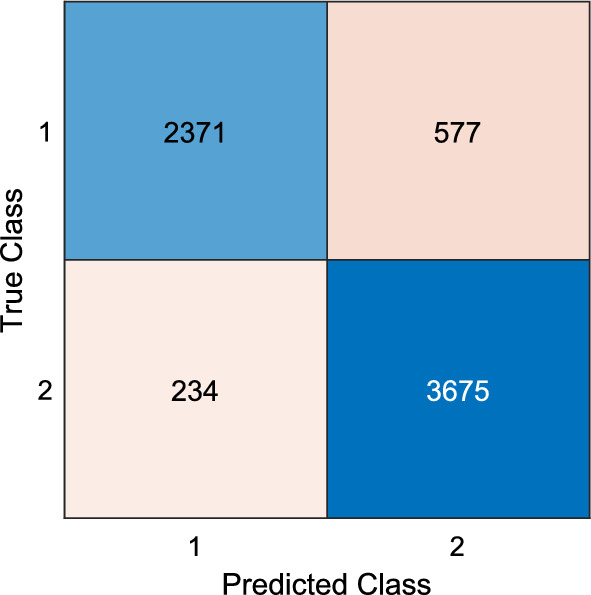
Confusion matrix of the developed model. **1: Alzheimer’s disease; 2: Control

With information fusion post-processing, the overall best model results of 88.17% accuracy and 86.96% geometric mean were observed at the 14th iteration (majority voted using the top 16 channel-wise results) (Table 4), which surpassed by large 10.02 and 9.50% margins the corresponding best channel-wise accuracy and geometric mean results (Table 3), respectively. The overall best model performance is depicted as a confusion matrix in Fig. 4, which yields precision and recall values of 91.02 and 80.43%, respectively.

**Table 4:** Voted results (%) obtained using the GHPat-based AD detection model

n	Accuracy	Geometric mean	n	Accuracy	Geometric mean
3	82.67	81.30	12	87.72	86.49
4	83.67	83.63	13	86.67	84.71
5	85.07	83.43	14	87.72	86.54
6	86.04	85.70	15	87.20	85.25
7	86.31	84.59	16	**88.17**	**86.96**
8	87.57	86.85	17	87.22	85.29
9	86.19	84.11	18	88.13	86.82
10	87.34	86.32	19	87.31	85.28
11	86.57	84.71	20	87.71	86.13

^**^n, number of the top channels used in the iterative majority voting

**Fig. 5:**
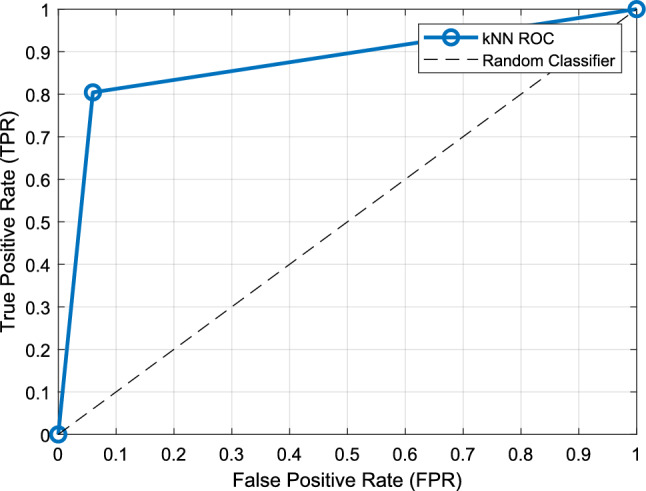
ROC curve for the overall best model

Figure 4 shows the confusion matrix of the model with the highest classification performance. The performance metric values and ROC curve calculated using this matrix are given in Table 5 and Fig. 5, respectively.

**Table 5:** Overall best model performance metric values

Metric	Value (%)
Accuracy	88.17
Precision	91.02
Recall	80.43
Geometric mean	86.96
F1 Score	85.40
Cohen’s Kappa	75.52
Area under curve	87.22

## Discussions

**Fig. 6:**
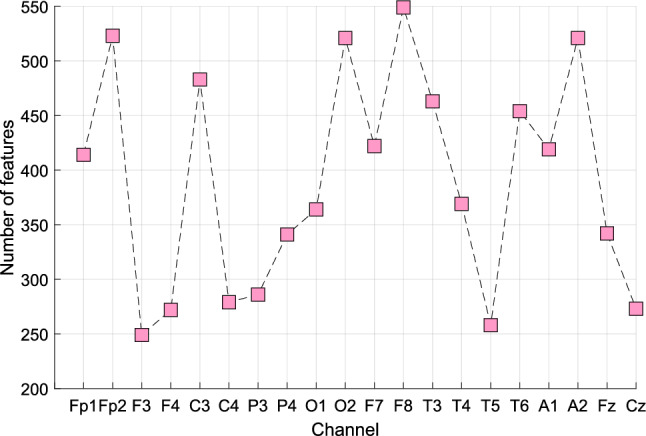
Lengths of selected feature vectors in various individual EEG channels

Our feature engineering architecture encompasses four key stages: (1) multilevel hybrid feature extraction, (2) feature selection, (3) classification, and (4) post-processing. In Stage 1, 2160 features were extracted from each of the 20 EEG channels. From these, INCA generated selected feature vectors with varying optimal lengths (in the iteration range of 50–550). In our experiments on the study dataset, the longest and shortest selected feature vectors were in Channels F8 (length 549) and F3 (length 249), respectively (Fig. 6).

**Fig. 7:**
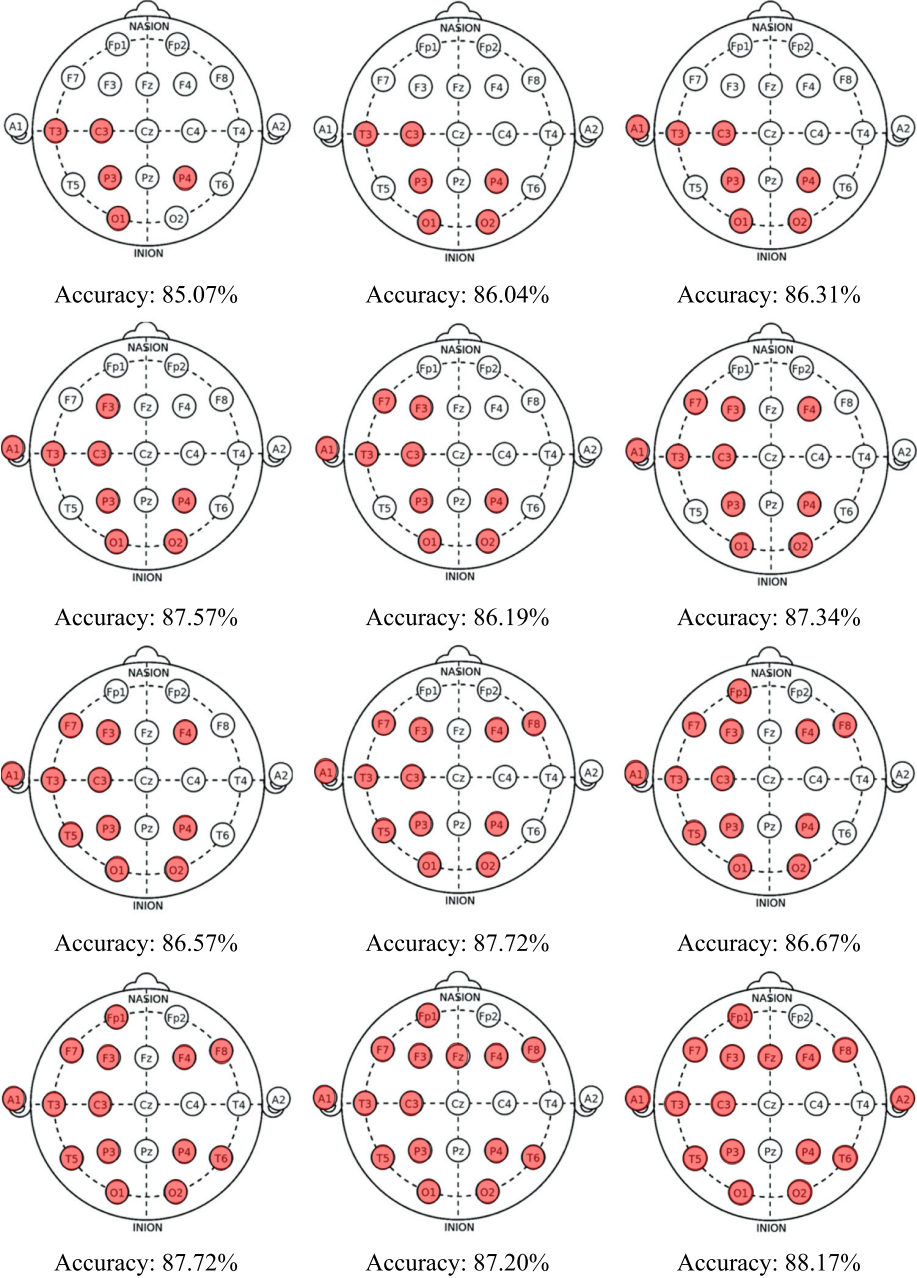
Model accuracy obtained and the corresponding computed cortex maps showing the numbers and placement sites of scalp EEG electrodes (pink circles) that contributed signal inputs to the accuracy evaluations. The cortex maps are shown from left to right, and top to bottom, in ascending order of increasing numbers of active contributing scalp EEG electrode inputs. The highest post-processed accuracy result of 88.17% was obtained with 16 scalp electrodes, as depicted on the right of the last row

Upon application of information fusion post-processing, classification accuracy improved substantially via a self-organized selection of the best overall result constituted from the top 16 IMV-selected channel-wise results to the exclusion of the T4, Fp2, Cz, and C4 channels. Moreover, we generated cortex maps to facilitate visual correlation of the overall model accuracies with the EEG channels that had contributed EEG signal inputs to the performance evaluations (Fig. 7). Interestingly, in the cortex maps involving smaller numbers of the top contributing EEG channels, the bilateral parietal leads, P3 and P4, appear to predominate (Fig. 7, top row). While the underlying pathological basis of this observation remains to be elucidated, the cortex maps offer an element of explainability to the model results.

**Fig. 8:**
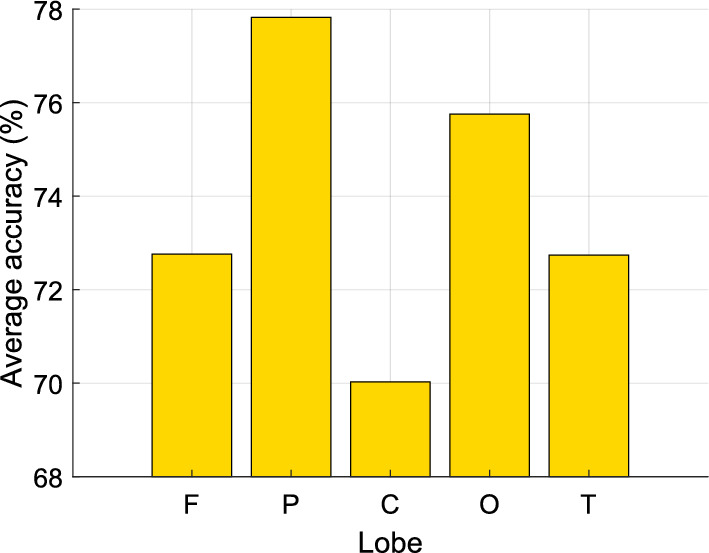
Average classification accuracies obtained for various brain regions using our proposed model. **C, central; F, frontal; O, occipital; P, parietal; T, temporal

To evaluate the relative contributions of various brain regions to model performance, we grouped the channel-wise results according to brain regions and computed the average classification accuracies for each region (Fig. 8). The highest and lowest contributors to model accuracy were EEG channels placed in the parietal (average classification accuracy 77.83%) and central (average classification accuracy 70.03%) brain regions, respectively.

### Neurological discussion of the findings

Our model results suggested the parietal lobe as the most affected region in AD, providing interesting insights into clinicopathological correlations. The neocortex, entorhinal cortex, and hippocampus within the temporal lobe are traditionally considered the pathophysiological starting points for structural and functional perturbations in AD. In contrast, using metabolic imaging techniques such as PET, SPECT, and functional MRI, the pathophysiology is more likely to be originating from the temporoparietal junction, medial parietal region, and posterior association areas, whereas the medial temporal region appears to be less affected (Jacobs et al. 2012). In our study, we observed that the most affected channels were P4 and P3 (Table 3, Fig. 7), which is consistent with metabolic imaging observations (Jacobs et al. 2012). Another finding from our study is that the most affected channels were P4, P3, C3, T3, T5, O1, and O2 (Table 3). The proximity of these channels is in line with the known behavior of AD pathology spreading to neighboring (Braak and Braak 1996; Thal et al. 2002). Some studies have reported differential impacts on the right and left hemispheres in AD patients, while others have found no asymmetry between the hemispheres (Jacobs et al. 2012; Foundas et al. 1997). In our study, we did not observe any hemispheric dominance (Table 3). We observed the temporal, parietal, and occipital areas to exhibit more pronounced effects compared to the frontal regions (Table 3, Fig. 7), which is consistent with results obtained from metabolic imaging (Foster et al. 1984). In light of our findings, analysis of EEG data using appropriate methods holds promise not only for diagnostic screening of AD but also potentially for staging the severity of AD.

### Ablation studies

We performed ablation studies to focus on individual model elements on a simplified base model. To enhance the sensitivity of the analysis, we evaluated the performance of the best-performing lead, P4 (Table 3), using a tenfold CV (which is less rigorous and more forgiving than the LOSO CV used in our primary analysis) with a kNN classifier across all cases. The ablation study cases are listed below.

*Case 1:* Feature extraction using only GHPat.

*Case 2:* Feature extraction using only statistical moments.

*Case 3:* Feature extraction using both GHPat and statistical moments.

*Case 4:* Feature extraction using standard LBP method.

*Case 5:* Full feature engineering model.

**Fig. 9:**
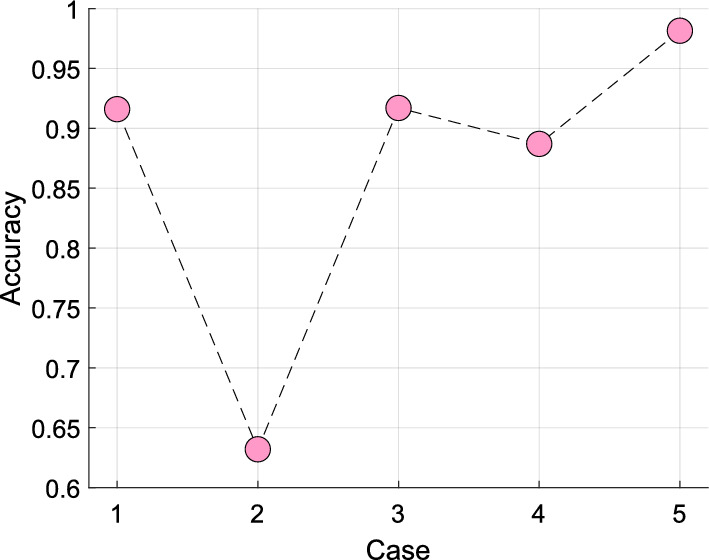
Single-channel P4 classification accuracies obtained for various cases with tenfold cross-validation and kNN classifier

With this analysis, our model (Case 5) attained the best classification accuracy of 98.15% versus other cases (Fig. 9). Of note, statistical features attained the worst classification accuracy of 63.22%, underperforming LBP (88.73% accuracy); and hardly improving the performance of combined GHPat-based textural feature and statistical feature extraction (91.69% accuracy) compared with GHPat feature extraction alone (91.61% accuracy).

### Comparative results

To compare our proposed model with published feature engineering models for EEG-based AD detection, we performed a non-systematic review. To facilitate comparison, we also appended our results analyzed using a tenfold CV, the more widely reported performance metric used in the literature (Table 6). Of note, the current study used the largest AD EEG dataset. Based on k-fold CV, our model outperformed most of the reviewed studies for binary classification into AD and non-AD classes. Dogan et al. (Dogan et al. 2022) obtained a perfect tenfold CV classification accuracy in their study, but the number of subjects used in the study was comparatively low.

**Table 6:** Comparison of the GHPat-based model with recently published machine learning models

Authors	Dataset	Method	Classification	Validation	Results (%)
Rodrigues et al. (2021)	4 classes (11 HC, 8 MCI, 11 MAD, 8 AD)	DWT, cepstral and lacstral analysis, feature normalization, and artificial neural network	HC vs. MCI vs. MAD vs. AD	LOO CV	Acc 95.55 Sen 90.83 Spe 97.73 AUC 94.7
Araújo et al. (2022)	3 classes (11 HC, 8 MCI, 8 AD)	Wavelet packet decomposition, entropy-based non-linear analysis, F-score selection, and SVM	HC vs. MCI vs. AD	LOO CV	Acc 56.8
Ruiz-Gómez et al. (2018)	3 classes (37 AD, 37 MCI, 37 HC)	MLP	HC vs. AD + MCI	LOSO CV	Sen 82.35
Oltu et al. (2021)	3 classes (16 MCI, 8 AD, 11 HC)	Bagged Trees	MCI vs. AD vs. HC	fivefold CV	Acc 96.50 Sen 96.21 Spe 97.96
Fiscon et al. (2014)	3 classes (100 participants AD, MCI, CT)	SVM, J48 and DMB	AD vs. CT + MCI vs. CT + AD vs. MCI	86-fold CV	Acc 90.00 Spe 87.00
Fouladi et al. (2022)	3 classes (63 AD, 56 MCI, 61 HC)	Modified CNN, Conv-AE	AD vs. MCI vs. HC	fivefold CV	Acc 92.00
Ieracitano et al. (2020)	3 classes, 189 participants (63 AD, 63 MCI, 63 HC)	MLP, SVM, AE, LR	AD vs. HC + AD vs. MCI + MCI vs. HC + AD vs. MCI vs. HC	tenfold CV	Acc 96.95
Kachare et al. (2024)	2 classes (12 AD, 11 HC)	Lightweight CNN	HC vs. AD	60:20:20	Acc: 98.50 Sen: 100.0 Spe: 97.55
Puri et al. (2024)	2 classes (12 AD, 11 HC)	Low-complexity CNN	HC vs. AD	70:10:20	Acc: 99.24 Sen: 100.0 Spe: 98.18
Sharma and Meena (2025)	2 classes (80 AD, 12 HC)	Graph wavelet transform, statistical features	HC vs. AD	80:20	Acc: 99.75
Stefanou et al. (2025)	3 classes (36 AD, 23 FD, 29 HC)	CNN	AD + FD vs HC	LNSO	Acc: 80.69
kumar Ravikanti D, Saravanan S, (2023)	3 classes (86 AD/MCI, 23 HC)	Wild Geese Migration Optimization Algorithm, CNN	AD + MCI vs HC	75:25	Acc: 96.18 Sen: 96.33 F1: 96.17
Devi and Latha (2025)	3 classes (13 AD, 7 MCI, 15 HC)	Gazelle Optimization Algorithm, CNN, Improved Tuneable Q Wavelet Transform	Unspecified	Unspecified	Acc: 97.25 Spe: 94.65
Puri et al. (2025a)	2 classes (12 AD, 11 HC)	ReptileSearch Algorithm and Snake Optimizer	AD vs. HC	Unspecified	Acc: 99.22 Sen: 99.68 Spe: 98.23
Joshi et al. (2025)	3 classes (59 AD, 7 MCI, 102 HC)	Multilayer perceptron, bidirectional LSTM	MCI vs. AD vs. HC	fivefold CV	Acc: 97.27
Puri et al. (2025b)	3 classes (59 AD, 7 MCI, 102 HC)	Higuchi’s fractal dimension, Hjorth parameters, DWT	AD vs. MCI vs. HC	tenfold CV	Acc: 98.65
Acharya et al. (2025)	3 classes (36 AD, 23 FD, 29 HC)	CNN	HC vs. FD vs. AD	80:20	Acc: 95.70
Bedoin et al. (xxxx)	2 classes (46 AD, 32 SCI)	Phase-lag index, dynamic time warping	AD vs. SCI	tenfold CV	AUC: 98.70
Chen et al. (2025)	3 classes (100 AD, MCI, HC)	Integrated multimodal learning	AD vs. HC	80:20	Acc: 90.00 F1: 88.57
Lee et al. (2025)	3 classes (30 AD, 30 MCI, 30 HC)	Statistical analysis	AD vs. MCI vs. HC	Unspecified	Acc: 92.00
Our model	2 classes (113 non-AD, 134 AD)	Multilevel DWT, GH graph, INCA, kNN, and IMV	Non-AD vs. AD	tenfold CV LOSO CV	tenfold CV Acc 99.85 Gm 99.84
					LOSO CV Acc 88.17 Gm 86.96

^**^Acc: accuracy; AD: Alzheimer’s disease; FD: Frontotemporal Dementia; AUC: area-under-curve; CV: cross-validation; DWT: discrete wavelet transform; GH: Goldner-Harary; Gm: geometric mean; HC: healthy control; INCA: iterative neighborhood component analysis; IMV: iterative majority voting; kNN: k-nearest neighbors; LOO: leave-one-out; LOSO: leave-one-subject-out; MCI: mild cognitive impairment; MAD: mild Alzheimer’s disease; Sen, sensitivity; Spe, specificity; SVM: support vector machine; MLP: multi-layer perceptron; J48, decision tree; DMB: data mining big; Conv-AE: convolutional autoencoder; AE: autoencoder; LR: Logistic Regression. LSTM: long short-term memory, LNSO: Leave-N-subjects-out, SCI: Subjective Cognitive Impairment

### Limitations

Limitations of our study:

1. The dataset used in this study is specific to a certain population, which may limit the model’s generalizability. Testing the model on larger and more diverse EEG datasets, including different stages of Alzheimer’s disease and various demographic groups, could improve its robustness. To achieve high classification performances, the most of the researchers have used deep learning architectures and these architectures are computationally expensive. Therefore, lightweight and accurate models should be presented for EEG signal classification.
2. The Goldner-Harary graph subpattern-based feature extraction method introduces a new approach to EEG signal processing. Future research could explore its applicability to other neurological disorders and further investigate its interpretability in clinical settings.

## Highlights

Highlights of our study include:

1. We trained our model on a large EEG AD dataset comprising data from 247 subjects, which supports the generalizability of our results.
2. We introduced a novel feature extraction function, GHPat, which fused feedforward networks with handcrafted graph-based feature extraction.
3. A self-organized architecture was developed for AD screening that attained 88.17% and 99.85% classification accuracies with LOSO and tenfold CV strategies, respectively.
4. Among channel-wise results, the P4 channel in the parietal region yielded the most accurate results. Analysis of model performance by brain regions also showed the parietal lobe to exert the most impact. These observations affirm the differential importance of various brain regions in AD pathogenesis and diagnosis.
5. Computed cortex maps enable visualization of brain regions affected by AD, and provide explainability to our results.
6. Comparative evaluations confirm the excellent classification performance of our GHPat over recently published machine learning models for AD detection.

In future works, we plan to expand our research by collecting a larger EEG signal dataset, with more participants in multiple categories. Additionally, we aim to develop next-generation quantum-inspired graph patterns using more complex graphs. Furthermore, we intend to explore the potential of deep learning models based on quantum patterns, such as convolutional neural networks, to further refine our classification capabilities.

## Conclusions

In this study, we present a novel feature engineering model that combined quantum-inspired GHPat feature extraction with a self-organized architecture for the detection of AD using EEG signals. Our model attained high classification accuracies of 88.17% and 99.85% with LOSO and tenfold CV strategies, respectively, which outperformed the literature, including the widely adopted 1D LBP feature extractor. Accuracy results stratified by channel placement and brain regions suggest P4 and the parietal region to be the most impactful and these channel-based results provided valuable findings into clinicopathological correlations. This work contributes to the burgeoning knowledge in AD detection utilizing EEG signals. Our proposed GHPat model is also computationally lightweight and accurate. Therefore, the recommended GHPat feature engineering is ready for using physical enviroment for EEG signal classification.

## Data Availability

The data presented in this study are available on request from the corresponding author. The data are not publicly available due to restrictions regarding the Ethical Committee Institution.
